# Potential Adverse Effects of Resveratrol: A Literature Review

**DOI:** 10.3390/ijms21062084

**Published:** 2020-03-18

**Authors:** Abdullah Shaito, Anna Maria Posadino, Nadin Younes, Hiba Hasan, Sarah Halabi, Dalal Alhababi, Anjud Al-Mohannadi, Wael M Abdel-Rahman, Ali H. Eid, Gheyath K. Nasrallah, Gianfranco Pintus

**Affiliations:** 1 Department of Biological and Chemical Sciences, Lebanese International University, 1105 Beirut, Lebanon; abdallah.shaito@liu.edu.lb; 2 Department of Biomedical Sciences, University of Sassari, 07100 Sassari, Italy; posadino@uniss.it; 3 Department of Biomedical Science, College of Health Sciences, and Biomedical Research Center Qatar University, P.O Box 2713 Doha, Qatar; ny1204022@student.qu.edu.qa (N.Y.); Dalhababi@hmc.org.qa (D.A.); aalmohannadi2@sidra.org (A.A.-M.); 4 Institute of Anatomy and Cell Biology, Justus-Liebig-University Giessen, 35392 Giessen, Germany; hibahasan145@gmail.com; 5 Biology Department, Faculty of Arts and Sciences, American University of Beirut, 1105 Beirut, Lebanon; sarahhalabi5@gmail.com; 6 Department of Medical Laboratory Sciences, College of Health Sciences and Sharjah Institute for Medical Research, University of Sharjah, Sharjah P.O Box: 27272, United Arab Emirates; whassan@sharjah.ac.ae; 7 Department of Pharmacology and Toxicology, Faculty of Medicine, American University of Beirut, P.O. Box 11-0236 Beirut, Lebanon

**Keywords:** resveratrol, biphasic, anticancer, reactive oxygen species (ROS), oxidative DNA damage, antioxidant effects, pro-oxidant effects

## Abstract

Due to its health benefits, resveratrol (RE) is one of the most researched natural polyphenols. Resveratrol’s health benefits were first highlighted in the early 1990s in the French paradox study, which opened extensive research activity into this compound. Ever since, several pharmacological activities including antioxidant, anti-aging, anti-inflammatory, anti-cancerous, anti-diabetic, cardioprotective, and neuroprotective properties, were attributed to RE. However, results from the available human clinical trials were controversial concerning the protective effects of RE against diseases and their sequelae. The reason for these conflicting findings is varied but differences in the characteristics of the enrolled patients, RE doses used, and duration of RE supplementation were proposed, at least in part, as possible causes. In particular, the optimal RE dosage capable of maximizing its health benefits without raising toxicity issues remains an area of extensive research. In this context, while there is a consistent body of literature on the protective effects of RE against diseases, there are relatively few reports investigating its possible toxicity. Indeed, toxicity and adverse effects were reported following consumption of RE; therefore, extensive future studies on the long-term effects, as well as the in vivo adverse effects, of RE supplementation in humans are needed. Furthermore, data on the interactions of RE when combined with other therapies are still lacking, as well as results related to its absorption and bioavailability in the human body. In this review, we collect and summarize the available literature about RE toxicity and side effects. In this process, we analyze in vitro and in vivo studies that have addressed this stilbenoid. These studies suggest that RE still has an unexplored side. Finally, we discuss the new delivery methods that are being employed to overcome the low bioavailability of RE.

## 1. Introduction

Resveratrol (RE; (3,4’,5 trihydoxystilbene)) is a stilbenoid natural polyphenol. RE was first isolated in 1939 by Takaoka from *Veratrum grandiflorum* [1,2]. RE is found in over 70 plant species but is highly concentrated in the skin of red grapes. Tea, berries, pomegranates, nuts, blueberries, and dark chocolate are also reported to contain RE at varying concentrations.

Resveratrol exists as two isomeric forms (*cis* and *trans*), yet the *trans* form is the predominant form and it has the most potent therapeutic benefits owing to the lower steric hindrance of its side chains [3,4]. The *trans* form can be recombinantly obtained from the extracts of yeast (*Saccharomyces cerevisiae*) and is used in the industry as a food supplement or as a cosmetic ingredient [5,6]. Isomerization to the *cis* form can occur when the *trans* form is exposed to heat, light, or ultraviolet radiation [7,8].

Resveratrol was reported to exhibit a plethora of therapeutic benefits, including anti-inflammatory, antioxidant, anti-platelet, anti-hyperlipidemic, immuno-modulator, anti-carcinogenic, cardioprotective, vasorelaxant, and neuroprotective effects [9,10] [11,12,13]. Indeed, RE was reported to be able to maintain or enhance human cerebrovascular functions [14], modulate in vitro angiogenesis through the expression of vascular endothelial growth factor (VEGF) and the formation of new vascular networks [15], stimulate human immune cell functions [16], promote rat cell viability and proliferation [17], ameliorate mitochondrial respiratory dysfunction, and enhance cellular reprogramming in human fibroblasts derived from patients with a mitochondrial disease [18], a phenomenon potentially mediated by the activation of Sirtuins [19]. Resveratrol has also showed proven cardioprotective [20,21], hepatoprotective [22], and neuroprotective activities [23]. In particular, this polyphenol seems to alleviate the main risk factors of cardiovascular diseases (CVD) as it can improve endothelial function, scavenge reactive oxygen species (ROS), reduce inflammation, inhibit platelet aggregation, and ameliorate the lipid profile and other main factors that can promote atherosclerosis [24,25]. Furthermore, redox-associated mechanisms were implicated as potential pathways via which RE elicits its cardioprotective effects. These redox-associated mechanisms include preservation of mitochondrial function under hypoxia/reoxygenation-induced oxidative stress [26], upregulation of antioxidant enzymes such as peroxidase and superoxide dismutase (SOD) [27], and modulation of nitric oxide (NO) production [28]. 

Although in vitro, ex vivo, and animal studies have indicated that RE may exert several health benefits and cardiovascular protection, in particular [20,21,29], the human clinical studies available so far have shown controversial results concerning the protective effects of RE against diseases and their sequelae [30,31,32,33,34,35,36]. The reasons behind these conflicting findings is varied; however, differences in the characteristics of the enrolled patients, RE doses used, and the duration of RE supplementation were proposed, at least in part, as possible causes [30,37]. In particular, the optimal RE dosage capable of maximizing RE health benefits without raising toxicity issues remains to be elucidated and is an area of extensive research [31,33,38]. 

Despite its toxicity may appear controversial, the dose of RE, as well as its interaction with the redox state of the environment where it is present can determine, to a large extent, whether it will exert beneficial or deleterious effects [33,38,39,40,41,42,43]. Moreover, the so-called hormetic property of RE may also be responsible for several controversial results associated with this molecule [44,45]. Hormesis refers to the bidirectional (biphasic) responses of a cell/organism to a chemical or other external stressors and is characterized by stimulation at low doses (usually associated with beneficial effects) and an inhibition by high doses (usually a toxic effects) [46]. Many of the RE-elicited dose-dependent responses, in vitro and in vivo, lead to positive responses at low doses and negative responses at high doses and, hence, may be explained by a hormetic dose–response effect [44], 

Currently, RE hormetic effects are a subject of controversy. Resveratrol appears to have a different effective dosage range in vitro (micromolar range in cell culture media) than its in vivo bioavailability (nanomolar range in the blood), thus making it difficult to identify the actual biologically effective concentration range at which this compound should be supplemented to human subjects. In this regard, concerns were raised regarding the ability of attaining the in vitro effective concentrations in vivo. We suggest that the actual biologically effective concentration range of *RE in vivo* remains to be determined. While the actual levels of RE in organs and tissue of humans remain under investigation, multiple lines of evidence indicate that, in rodents, RE can accumulate in specific tissues or organs at relatively high concentrations, that are comparable to those used in many in vitro experiments. For instance, RE plasma peak concentrations of 32 µM were reported in rodents [47,48]. Moreover, after chronic consumption, RE was detectable in plasma up to one week after wash-out [49]. Because of the lipophilic nature of most natural antioxidants, their levels in tissue, which outlast their presence in the plasma, may provide a better indicator of the in vivo biologically active concentrations of RE. Indeed, concentrations of RE in tissues such as the heart, liver, and kidney were higher (~10–30 µM) than in plasma in rats fed dietary-relevant doses of RE [50,51]. Although affected by a large degree of interindividual variability, a recent report indicated that picomolar concentrations of RE can accumulate in the colon cancer tissue of humans supplemented with dietary (5 mg) or pharmacological doses (1 g) of RE for few days [52]. However, the same authors reported that, after one hour, the plasma RE concentration could reach 137 µM in the human subjects receiving the pharmacological dosage [52].

Furthermore, it was suggested that plasma proteins may act as in vivo natural reservoirs for antioxidants. As a result, plasma proteins can modulate the plasma concentrations and tissue delivery of antioxidants [53,54,55,56]. Moreover, interactions between different natural antioxidants may also influence their kinetics and metabolism in the liver leading to an increase in the circulating levels of natural antioxidants [57]. Interestingly, studies have indicated that the half-life and plasma concentrations of RE metabolites is 10 times higher than that of the native RE compound [58]. Whether these metabolites can serve as a pool from which free RE can be locally released at various tissues cannot be excluded at the moment. 

**Figure 1:**
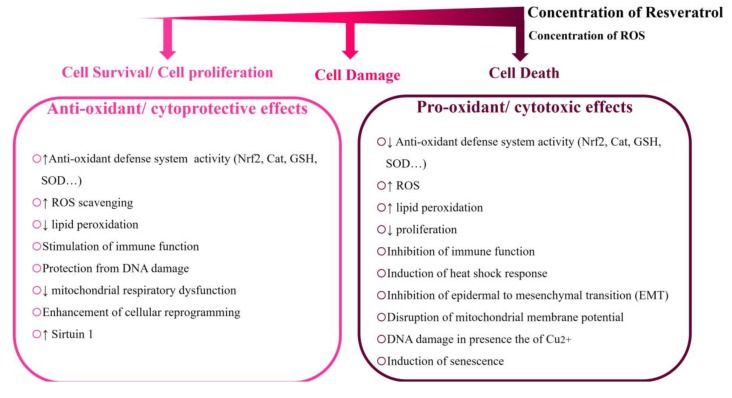
Biphasic hormetic dose-dependent effects of resveratrol (RE). Resveratrol exhibits biphasic dose-dependent effects. At low concentrations, RE acts as an antioxidant which can protect from DNA damage and oxidative stress. On the other hand, at high concentrations, RE acts as a pro-oxidant promoting DNA damage while increasing oxidative stress. Low and high concentrations offer beneficial effects in the prevention of cancer formation (chemo-preventive) and in the treatment of cancer (cytotoxic), respectively.

Although many studies have indicated that RE is a well-tolerated and safe compound in humans [59,60], others have reported toxic effects of RE in vitro and in vivo [44]. For example, RE exhibited systemic inhibition of P450 cytochromes, when taken in high doses. In addition, RE was shown to interact with several drugs. These interactions are harmful since, in most cases, they could attenuate the activities of these drugs [2]. Additionally, long-term intake of RE can act as a thyroid disruptor and a goitrogen [61,62], not to mention all the toxic side effects related to RE high-dosage-associated hormetic effects in vitro and in vivo [44,63,64], including the high-dose-associated pro-oxidant effects [36,38,39,43,64,65] (Figure 1).

## 2. Resveratrol Has Poor In Vivo Pharmacokinetics

The commercial use of RE as a pharmaceutical drug is currently facing several limitations; in particular, its low bioavailability and rapid metabolism are addressed as some of the most limiting. In this regard, the in vivo effects of RE appear to be affected by its low solubility and low bioavailability [66]. Oral intake of 25 mg of RE revealed that it has extremely low bioavailability, where only trace amounts (<5.0 ng/mL) of un-metabolized RE could be detected in the plasma [66]. After consumption, more than 70% of RE is absorbed by the gastrointestinal tract, but it is later metabolized by three distinct metabolic pathways leading to its very low bioavailability. Extremely rapid sulfate conjugation of RE in the intestine/liver looks to be the rate-limiting factor in determining the bioavailability of RE [66].

RE also exhibits low water solubility (<0.05 mg/mL), which affects its absorption. Both RE stability and solubility are strongly influenced by pH and temperature [67]. In this context, Zupančič et al. revealed that RE solubility at pH 1.2 is 64 μg/mL, while it becomes 61 and 50 μg/mL at pH 6.8 and above pH 7.4, respectively. The same authors also reported that, once solubilized in water, RE is stable at room or body temperature only under acidic conditions; however, with increasing pH, the stilbene is degraded exponentially. It appears that RE is most stable, in liquid form, at low pH and temperature and at limited exposure to oxygen and light [67].

**Figure 2:**
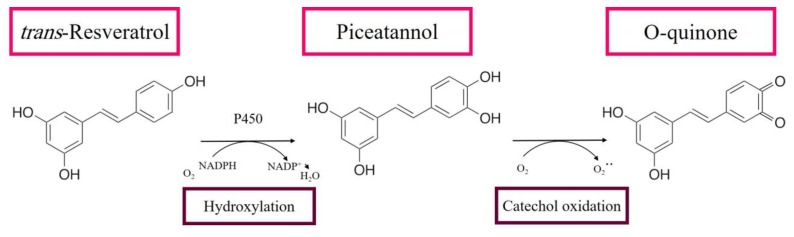
Metabolism of resveratrol in the liver.

Following oral intake, RE undergoes passive diffusion or can form complexes with transporters such as integrins, albumin, and low-density lipoprotein (LDL) [7,68,69]. Under the acidic environment of the stomach, RE appears to be stable, but it can be hydrolyzed to oligomeric phenolics and/or be subjected to isomeric conversion. Furthermore, RE glycosylation by gut-resident bacteria can give rise to piceid, which is a stilbenoid glucoside (resveratrol-3-*O*-beta-glucoside) that can be absorbed in the intestine [70]. Resveratrol modification can also occur via intestinal and hepatic conjugation reactions. Intestinal bacteria can break RE to benzoic, phenylacetic, and propionic acids, while, in the liver, it undergoes phase II metabolism producing glucuronidated, sulfated, and methylated products which are known to retain part of the biological activity of the compound of origin [7,68,69,71]. For instance, in the liver, RE is usually metabolized to piceatannol (Figure 2), which can be released into the bloodstream and can further give rise to piceatannol glucuronides or piceatannol sulfates that can return to the gut [72]. Interestingly, RE can also cause an increase of its own metabolism by enhancing the activity of phase II hepatic detoxifying enzymes [73].

Despite its controversial low bioavailability and rapid metabolism, there are many reports about a multitude of RE in vivo biological effects [44,58]. In this regard, the in vivo biological effects of RE are also related to its affinity to transport proteins. It was extensively reported that RE can form complexes with human serum albumin (HSA) and lipoproteins, an interaction that improves RE stability and functioning since the plasma transport proteins can act as in vivo natural reservoirs of RE [56,74,75,76,77,78]. Resveratrol–HSA or RE–LDL complexes facilitate RE entry into different tissues [66,68] In the bloodstream, HSA is crucial for binding, transporting, promoting cellular absorption, and distributing RE to various cellular targets [78]. In this context, we previously reported that, under aqueous physiological conditions, HSA is able to bind and stabilize epigallocatechin gallate (EGCG), another biologically active natural antioxidant present in green tea. Therefore, HSA and other plasma proteins may be of primary importance in mediating the biological effects of RE, in vivo. Furthermore, RE is known to induce its own metabolism which increases the activity of phase II hepatic detoxifying enzymes, and resveratrol metabolites, including dihydro-resveratrol glucuronides, resveratrol glucuronides, and glucosides. These metabolites were found at high concentrations in human plasma and urine [70,79]. In this regard, half-life and plasma concentrations of RE metabolites in the blood were found to be 10 times higher than that of the native RE compound [58], suggesting that free RE may be locally released from these metabolites.

Yet, despite its low bioavailability and relatively rapid metabolism and elimination, RE shows a relevant biological efficacy, which may be due to its conversion/interconversion into sulfonate and glucuronide metabolites and/or its binding/unbinding to plasma proteins, two potential primary aspects in the delivery of RE at target organ sites [68,80]. 

## 3. Resveratrol Harmful Effects: Molecular Evidence

### 3.1. RE Metabolites Can Exhibit Cytotoxic Effects

Similar to RE, its metabolites can elicit a wide range of bioactivities. In general, metabolites of phenolic plant extracts can generate cytoprotective and beneficial effects or, on the other hand, can generate cytotoxic or immune-toxic effects [81]. *o*-Quinones are common reactive metabolites that can be formed via several metabolic mechanisms. In the case of RE metabolism, *o*-quinones are formed through a hydroxylation reaction by cytochrome P450 (Figure 2), leading to the formation of piceatannol, followed by catechol oxidation to form an *o*-quinone product. These RE metabolites could have different effects on several biological targets [81,82,83]. 

Piceatannol exhibits beneficial anti-inflammatory and antioxidant properties. Piceatannol inhibits prototypic tumor promoter-induced cyclooxygenase-2 (COX-2) and inducible nitric oxide synthase (iNOS) expression by blocking the activation of NF-κB (nuclear factor kappa-light-chain-enhancer of activated B cells) [84]. The Keap1–Nrf2 (kelch-like ECH-associated protein 1—nuclear factor erythroid 2–related factor 2) pathway, another target of piceatannol, can lead to the induction of detoxification enzymes. For instance, piceatannol enhances the expression of the antioxidant enzyme hemeoxygenase-1 (HO-1) in human mammary epithelial cells by induction of Nrf2 [85]. Piceatannol also prevents the activation of c-Jun N-terminal kinase (JNK) and downregulation of the anti-apoptotic B-cell lymphoma 2 protein (Bcl-2), which results in the inhibition of hydrogen peroxide- and peroxynitrite-induced apoptosis [86]. 

Beneficial effects caused by RE metabolites were also reported in vivo. In mice fed a high-fat diet, piceatannol could lower hepatic levels of tumor necrosis factor-alpha (TNF-α) and increase the expression of sirtuins, which are well-known players of cellular homeostasis [87]. By increasing the phosphorylated forms of adenosine 5’-monophosphate-activated protein kinase (pAMPK) and acetyl-CoA carboxylase (pACC) and by decreasing the protein levels of peroxisome proliferator-activated receptor γ (PPARγ) and fatty acid synthase (FAS), piceatannol could decrease the accumulation of lipids in adipocytes and in the liver, thus promoting an anti-obesity effect in mice fed a high-fat diet [88]. Furthermore, as evidenced by the reduction of potent inflammatory mediators such as interleukin-6 (IL-6) and monocyte chemoattractant protein-1 (MCP-1), RE could suppress inflammation in a mouse model of inflammatory edema [89]. In addition, piceatannol could inhibit the phosphorylation of p38 mitogen-activated protein kinase (p38-MAPK), leading to a decrease in the deposition of extracellular matrix proteins and amelioration of fibrosis in a mouse model of renal fibrosis [90]. 

*o*-Quinone metabolites of RE are associated with toxic effects, particularly in the skin. These toxic effects involve oxidative stress and alkylation mechanisms [91,92,93]. *o*-Quinone-induced inhibition of P450 oxidative enzymes or alkylation of certain proteins such as Keap1, Nrf2, I kappa B kinase (IKK), and NF-κB can also lead to hepatic and renal toxicity. In addition, *o*-quinones can deplete glutathione (GSH) and affect nicotinamide adenine dinucleotide phosphate oxidase (NOX) function, ultimately leading to the induction of oxidative stress [35,81]. 

Rhododendrol, a tyrosinase inhibitor used in lightening/whitening cosmetics, can increase the incidence of leukoderma skin toxicity. Resveratrol, similar to rhododendrol, is a *p*-substituted phenol that is rapidly converted to toxic *o*-quinones [94]. It was shown that RE can act as a substrate of tyrosinase, a key enzyme in the production of melanin, to produce reactive *o*-quinones [92,95]. Tyrosinase-generated *o*-quinones from RE metabolism can decay to produce oligomers, which act as pro-oxidants that cause melanocyte cytotoxicity, due to their ability to bind thiol-containing proteins. 

### 3.2. Resveratrol Cytotoxic Mechanisms Can Induce DNA Breaks

For years, the mainstream notion was that increased consumption of RE would lead to better scavenging of reactive oxygen species (ROS) and, therefore, RE may offer cytoprotective effects [96,97]. However, as highlighted in the introduction, under certain conditions, an antioxidant may act as a pro-oxidant, leading to acceleration of lipid peroxidation and/or induction of DNA damage. In fact, RE could have pro-oxidant activities, rather than antioxidant activities, depending on RE concentration, RE form, treatment conditions, and time of treatment, as well as the type of cells used and their basal redox state [39,98,99,100,101,102,103]. Interestingly, even chronobiology was shown to play a role in RE varying effects; when administered during the dark span, RE exerted an antioxidant effect by decreasing lipid peroxidation, whereas, during the light span, RE increased lipid peroxidation [39]. 

Whenever RE acts as a pro-oxidant molecule in vitro, it can cause DNA damage and reduce several DNA repair pathways, which can activate cytotoxic and apoptotic pathways [104,105]. The ability of RE to induce DNA breaks has a potential therapeutic use that can be harnessed when RE is used against cancerous cells. Copper levels are generally increased in various malignancies; this explains the preferential cytotoxicity of RE toward malignant cells in particular. In addition, electron transfer between RE and copper ions is higher in cancer cells [106,107]. Thus, RE- and copper-induced DNA damage may be one mechanism of RE cytotoxicity against cancer cells (Figure 2) [108]. Indeed, RE pro-oxidant effects are known to evoke a pro-apoptotic function in different types of cancerous cells [109]. 

RE’s effect can also be attributed to its ability to inhibit key enzymes critical for DNA synthesis such as ribonucleotide reductase and DNA polymerases [110,111,112]. In addition, resveratrol was reported to induce synthesis (S)-phase arrest and cellular senescence by modulating the chemokine receptor C-X-C motif chemokine receptor 2 (CXCR2)–p53 axis in U2OS and A549 cancer cells, as well as in normal human fibroblasts. Moreover, RE was reported to provoke DNA damage in colon cancer cells through topoisomerase II and activation of the ataxia-telangiectasia mutated (ATM) kinase to trigger p53-dependent apoptosis [113]. A significant increase in DNA double-strand breaks was found in RE-treated U2OS and A549 cancer cells. This phenomenon also appears to be mediated by RE-elicited pro-oxidant effects, as well as the modulation of the CXCR2–p53 pathway [114].

### 3.3. Resveratrol Cytotoxic Mechanisms Can Induce Oxidative Stress

Oxidative stress can be caused by a deficiency in the antioxidant defense system and an excess of pro-oxidants. Whereas antioxidants may delay or block apoptosis, increased oxidative stress is pivotal to overwhelm the cell and force it toward an apoptotic fate [115]. Reactive oxygen species/reactive nitrogen species (ROS/RNS) include both free radical species and non-free radical molecules. ROS include different species such as the hydroxyl radical (•OH), the most toxic species due to its extremely high reactivity [116] as well as superoxide (•O_2_), and hydrogen peroxide (H_2_O_2_), which are less reactive than •OH. RNS include an unreactive species, nitric oxide (NO•), and its derivative, peroxynitrite (ONOO^−^), a powerful oxidant which can destroy many biomolecules [117].

The ROS/RNS molecules usually have dual roles in both health and disease; ROS/RNS at low or moderate concentrations contribute to basic physiology such as blood pressure regulation, neurotransmission, and immune responses. However, excessive production of ROS/RNS can lead to oxidative/nitrosative stress and can result in deleterious alterations including cell death [118]. 

Several lines of evidence indicate that RE heavily influences the cellular redox state [36,38,39,40,41,42,43,63,64,65]. In this regard, low doses of RE have diverse beneficial actions, such as protecting cells and tissues against neurodegeneration, cardiovascular disease, cancer, diabetes, and obesity-related disorders and extending the lifespan of organisms [119,120]. This wide range of beneficial biological effects might be explained, at least in part, by RE’s antioxidant properties [31,121,122]. RE can also modulate NO release, which is crucial for endothelial function [123]. ROS decreases NO production and bioavailability [124], while RE increases them [125,126]. 

In addition, it was shown that RE can activate the Keap-1/Nrf2 antioxidant defense system in obese–asthmatic rats, thus protecting them against oxidative stress [60]. The RE-induced Nrf2 activity enhances the antioxidant defense system in rats with metabolic syndrome, evident by increased expression of catalase (CAT), SOD isoforms, peroxidases, glutathione-*S*-transferase, and glutathione reductase [127]. RE can also act as a potent antioxidant via the Nrf2/HO-1 signaling pathway, increasing SOD, glutathione peroxidase, and CAT activities and HO-1 protein levels, as well as decreasing lipid peroxidation in the brain tissue of RE-treated mice (Figure 1) [128]. 

Yet, such potent antioxidant activities exerted by RE are not consistently observed. As previously mentioned, RE can behave either as an antioxidant or pro-oxidant depending on several parameters including the dose and the microenvironment. Many studies demonstrated that RE has biphasic concentration-dependent effects, being an antioxidant at low doses and pro-oxidant at high doses both in vitro and in vivo [36,38,39,40,41,42,43,63,64,65]. It appears that RE pro-oxidant effects are usually followed by phospho-PKB/Akt (protein kinase B/ AKR mice thymoma) downregulation, cellular damage, and apoptosis. Interestingly, RE-induced pro-oxidant effects could be counteracted by *N*-acetyl cysteine (NAC) and diphenyleneiodonium (DPI), suggesting a role for flavin oxidases in pro-oxidant RE-induced toxicity [41]. Mitochondrial damage mediated by cytochrome P450 enzyme CYP2C9-produced ROS also appears to be involved in high-dosage-associated RE-elicited oxidative damage [42]. Moreover, RE was shown to affect male reproductive functions. Treatment with RE led to a dose-dependent reduction in the level of glutathione (GSH) with a concomitant increase in glutathione disulfide (GSSG), signifying an increased oxidative stress where a decreased glutathione/glutathione disulfide plasma ratio reflects increases in oxidative stress. Concomitantly, the activities of CAT and SOD were found to be decreased in a dose-dependent manner. This change in cellular redox amounted to a state of oxidative stress that eventually caused massive testicular tissue injury (Figure 1) [129].

**Figure 3:**
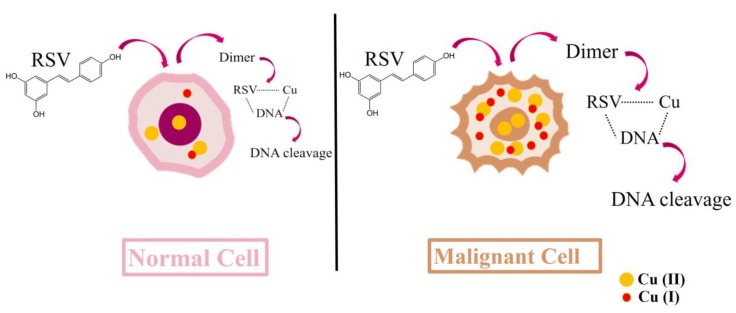
Resveratrol- and copper-induced cytotoxicity. The cytotoxic mechanisms of RE include the mobilization of endogenous copper ions, including chromatin-bound copper. Resveratrol undergoes oxidation in the presence of Cu(II) (which is substantially increased in the malignant cells) to a dimer. This electron transfer reduces Cu(II) to Cu(I). The dimer is capable of binding DNA to form a DNA–RE–Cu(II) ternary complex which allows the efficient cleavage of DNA. Considering that RE and copper-induced DNA damage will be considerably greater in cancer cells, this mechanism offers a way for the selective killing of cancer cells by using high concentrations of RE.

Interestingly, it was proposed that the pro-oxidant action of plant polyphenols, such as RE, could be a common mechanism for their cytotoxic properties that may inhibit the malignant phenotype of cancer cells [130,131]. While low doses of RE were reported to target the early stages of cancer (initiation and promotion), high concentrations of RE induce cell death by virtue of their pro-oxidant action. This offers a window of opportunity, which can be harnessed as a potential chemotherapy against several cancers (Figure 1 and Figure 3). Indeed, RE can lead to apoptosis and cell-cycle arrest of malignant melanoma cells [131]. Recently, it was also shown that RE induces caspase-dependent cell death in ovarian cancer cells via an ROS-dependent mechanism [132].

### 3.4. Resveratrol Suppresses the Expression and Activity of COX-1 and COX-2

Multiple lines of evidence suggest that the anti-inflammatory and chemo-preventive effects of RE are due to its ability to reduce expression and activity of COX-1 and COX-2 [133,134,135]. Indeed, RE inhibited the expression of COX-2 in lipopolysaccharide-treated Caco-2 cells, leading to a reduction of prostaglandin 2 (PGE_2_) production [136]. Non-steroidal anti-inflammatory drugs (NSAIDs), which inhibit COX-1 and COX-2, are heavily used in the management of inflammatory conditions, but they are not without adverse gastrointestinal side effects. Interestingly, similar effects were observed when RE was used to treat inflammatory conditions. Guha et al. demonstrated that RE treatment suppressed COX-1 expression and reduced the synthesis of PGE_2_ by gastric tissue. Effectively, RE delayed ulcer healing in mice with indomethacin-induced gastric ulcers [36].

### 3.5. Resveratrol Interacts with and Attenuates the Action of Other Drugs

Despite the considerable literature on RE, little is known about potential drug interactions with RE. A search of the clinical trials database (http://clinicaltrials.gov/) revealed that there are a total of 244 human clinical trials utilizing RE. These clinical trials investigated the potential beneficial effects of RE in the management of diabetes mellitus, obesity, Alzheimer’s disease, dyslipidemia, hypertension, stroke, cardiovascular diseases, kidney diseases, pulmonary diseases, eye diseases, rhinopharyngitis, inflammatory diseases, metabolic syndromes, and cancers [137]. RE was documented to exhibit adverse effects in most of these trials. It is becoming evident that RE interacts indirectly with other medications, leading to attenuation of the activity or overexpression of drug transporters and CYP450 enzymes, the major cellular system involved in drug metabolism [138]. Among the P450 enzymes, CYP3A4 is the main enzyme involved in the metabolism of over 50% of the marketed drugs that rely on metabolic elimination. Different studies suggest that RE alters or inhibits CYP3A4 enzyme activity [139,140]. 

Drug transporters, together with metabolic enzymes, are the main determinants that govern drug disposition. Previous studies indicated that RE could blunt the function and expression of drug transporters, thus improving the anti-proliferative activity and poor bioavailability of several drugs [141]. For instance, RE treatment can enhance the oral bioavailability of nicardipine, and this was associated with a decrease of P-glycoprotein-mediated efflux, with P-glycoprotein being a major drug transporter [142]. Moreover, it was shown (in vivo and in vitro) that RE enhanced methotrexate absorption in the intestine and decreased methotrexate renal elimination by inhibiting drug transporters that included P-glycoprotein, multidrug resistance-associated protein 2 (MRP2), and organic anion transporters (OAT1/OAT3) [143]. This RE effect may increase the risk of hepatotoxicity [144]. To add, RE can increase the anticoagulant activity of warfarin, which may increase the risk of bleeding [145].

RE co-treatment was also reported to attenuate the effects of several other drugs. For example, RE can attenuate the effects of Human immunodeficiency virus (HIV) protease inhibitors [146], and it can interact with 3-hydroxy-3-methylglutaryl coenzyme A reductase (HMG-CoA reductase) inhibitors [147], anti-arrhythmic agents [148], calcium channel agonists [149], antihistamines [150], and immunosuppressants [151].

## 4. In Vitro Evidence of the Harmful Effects Induced by RE

### 4.1. Resveratrol’s Concentration-Dependent Cytotoxicity in Different Cellular Models

RE can dose-dependently manipulate cellular oxidative stress levels and induce DNA damage, thus offering a potential therapeutic opportunity against cancerous cells (Figure 3). High concentrations of RE (50 µM) inhibited the proliferation of transformed macrophages, tumor-derived T cells, and epidermoid carcinoma cells. However, low concentrations of RE (5 µM) stimulated the proliferation of these cells. Similarly, low RE concentrations (0.5 µM–5 µM) did not alter viability or function of rat INS-1 pancreatic cells, while higher concentrations (50 µM) increased apoptotic cell death [152].

Resveratrol, at concentrations between 0.1 and 1 µM, elicited anti-proliferative effects in GRX cells, a cell culture model of activated hepatic stellate cells (HSCs) [153]. Indeed, high RE concentrations triggered a dose- and time-dependent rise of ROS, ultimately leading to cell death at a dosage of 50 µM of RE and higher [103]. in vitro, high doses of RE (50 µM) reduced GRX cell proliferation, while higher RE doses (70–100 µM) were cytotoxic to the cells [153]. This is consistent with findings showing that the high, but not low, RE concentrations (≥25 µM) induced the production of reduced glutathione and caused cell toxicity, in vitro [154]. 

By virtue of its ability to modulate oxidative stress, RE can sensitize cancer cells to chemotherapy. Indeed, the anti-cancer activity of paclitaxel was significantly enhanced when glioblastoma cells were treated with 50 μM of RE [155]. The potency of RE to inhibit malignant phenotypes can also be observed with high in vitro concentrations of RE (>50 μM) [114,156]. Indeed, RE induced senescence in osteosarcoma and lung carcinoma cells [114] (Figure 3). In addition, studies showed that RE exerts a genotoxic effect by inducing chromosomal aberrations, micronucleus cells, and polynuclear and karyorrhectic cells [157].

### 4.2. Resveratrol Alters the Redox State of Endothelial Human Cells

Alteration of the redox state of endothelial cells is a critical step in the onset and progression of CVD [158]. There is increasing interest in naturally occurring antioxidants and their particular impact on endothelial health. Contextually, RE appears to provide cardiovascular protection by virtue of its antioxidant impact on the endothelium [159,160]. Yet, several studies reported a dark side of RE. Posadino et al. reported that RE, at in vivo tissue-attainable doses, can increase the intracellular oxidative state. This caused mitochondrial membrane depolarization, provoked mitochondrial damage, and induced endothelial cell death [42]. Moreover, they showed that cytochrome P450 enzymes were the main source of oxidative stress induced by RE. They also demonstrated that RE exhibited a biphasic concentration-dependent effect on endothelial cells. Low in vitro RE concentrations (0.5 μM) exhibited antioxidant effects by decreasing endothelial cell oxidative state. However, higher in vitro concentrations of RE (≥10 μM and 25 μM) increased the endothelial cell oxidation state (Figure 1). Consistent with the observed pro-oxidant effects, increasing the dosage of RE exerted a significant decrease in metabolic activity of endothelial cells and their survival rate, which suggests a robust correlation between the pro-oxidant effect of RE and the cell damage observed [42]. In line, RE promoted the rapid increase in ROS levels, which resulted in a significant pro-oxidant activity [40] and apoptosis of endothelial cells [41].

### 4.3. Resveratrol Chemotherapeutic Doses Are Cytotoxic to Normal Healthy Cells

RE was demonstrated to decrease tumor volume, frequency, and incidence, as well as to increase tumor latency [161,162]. Molecularly, RE chemotherapeutic promise is thought to be achieved clinically by inducing oxidative stress and apoptosis in different types of cancer cells [109,114,163]. Yet, there were concerns about possible toxic effects exerted by the recommended chemotherapeutic doses of RE and other polyphenols on normal cells. The toxicity of different polyphenols, including RE, was studied on rat thymocytes. Among the different tested polyphenols, RE was the most cytotoxic to normal rat thymocytes. Resveratrol at 10 μM or higher led to a significant concentration-dependent increase in apoptotic cells [164]. RE also decreased the viability of 3T6 fibroblasts and HL60 tumor cells [165]. In mesenchymal stem cells, 0.1 μM RE promoted self-renewal, whereas concentrations above 5 mM increased senescence rate and inhibited self-renewal [166,167]. As such, further studies are needed to assert the safety of RE against normal healthy cell lines, especially before promoting RE as an anti-cancer agent. 

## 5. In Vivo Non-Human Evidence of the Harmful Effects of Resveratrol

RE is very well tolerated by experimental models with no major adverse effects. Orally administered RE, at doses of 200 mg/kg/day in rats and 600 mg/kg/day in dogs for 90 days, did not show any apparent side effect [168]. Yet, several studies reported that RE can exert toxic effects in experimental animal models.

### Resveratrol-Associated Toxicity in Rodents

Several studies reported the in vivo toxicity of RE in rats. For instance, when administered in high doses, RE could lead to death due to cardiac inflammation, dilatation of the renal tubules, necrosis of the papillary, acute inflammation in the pelvic area, and severe nephropathy. In addition, high doses of RE could cause significant increases in the blood urea nitrogen (BUN) and creatinine levels, as well as liver enzymes [35].

The cardioprotective effect of increasing doses of RE in parallel with another resveratrol formulation, namely, Longevinex, was also studied. Three different does of both RE and Longevinex (2.5 mg/kg, 25 mg/kg and 100 mg/kg) were administered to a group of rats for 30 days, while placebo was given to the control group. The data showed a hormetic response for pure RE, which provided cardioprotection at lower doses and detrimental effects at higher ones. Interestingly, Longevinex failed to display any hormetic action, remaining cardioprotective even at 100 mg/100 g body weight, a dose that induced 100% heart death when tested with pure resveratrol [63].

RE’s toxic effects were also studied in mice. A mouse model of indomethacin-induced gastric ulcers, a phenomenon where COX-1 and eNOS act as pivotal players in switching the RE biphasic effects form positive (healing ulcerative damage) to negative (promoting ulcerative damage), was employed. In this model, while a low dose of RE (2 mg∙kg^−1^) increased eNOS expression without affecting COX-1 expression, a high dose of RE (10 mg∙kg^−1^) significantly suppressed COX-1 expression, ultimately reducing PGE_2_ synthesis and the reparative angiogenesis in ulcerated mice. Administration of l-arginine before RE significantly increased tissue NO synthesis and improved ulcerative healing, confirming the RE high-dose pro-oxidant effect as responsible for promoting ulcerative damage [36]. 

A role for RE in kidney fibrosis was also studied in mice. in vivo, low-dose RE administration (≤25 mg/kg) partly improved renal function in mice with kidney damage caused by unilateral ureteral obstruction (UUO); high-dose administration of RE (≥50 mg/kg) lost its anti-fibrotic effect, aggravating renal fibrosis instead. Noteworthy, mice with kidney damage caused by unilateral UUO were more susceptible to high-dose RE-induced renal injury than normal mice [169]. 

The potential toxicity and anti-angiogenic activity of RE was also evaluated in zebrafish. Different doses of RE (10, 50, and 100 μM), considered relatively high in in vitro models, were used to treat zebrafish embryos. RE (100 μM) inhibited the formation of major blood vessels by downregulating VEGF. This dose-dependent toxicity was concomitant with teratogenic deformities, reduction in the survival rate, heart edema, and reduction in the hatching rate [170]. The lethal dose 50 (LD_50_) of zebrafish embryos treated with RE for 96 hours was 75.3 mg/L [171]. Moreover, the short-term in vivo chorionic ecotoxicity of zebrafish treated with RE showed an LD_50_ of 51.4 mg/L [171].

Given the reports about the toxicity of RE in rodents and zebrafish, combined with the lack of full data on RE systemic toxicity in other species, the toxicity of RE on target organs remains mostly not well defined and is pending further studies.

## 6. In Vivo and In Vitro Human Evidence of the Harmful Effects of Resveratrol

### 6.1. Resveratrol Can Lead to Hypersensitivity and Alteration of Human Cytokine, Blood, and Liver Parameters

Currently, RE supplementation is widely used in humans, because of its reported potential antitumor and anti-inflammatory properties; however, its harmful effects are not well characterized.

Here, we stress that RE doses that are considered high and toxic in vitro (above 25 µM) may not be high in vivo and that the optimal effective dose for human supplementation remains to be determined. Human subjects are yet to show any adverse effects following their supplementation with high doses of RE [172]. Nonetheless, Cottart et al. confirmed that, despite the extensive research on the benefits derived from RE, there was not enough research conducted to assess its harmful effects [59], especially since human clinical studies are limited. For instance, RE can cause considerable reductions in white blood cell (WBC) counts and plasma IL-6 or TNF levels, as well as elevations of plasma alanine aminotransferase (ALT) levels [173,174]. In addition, high doses of RE (2–5 g per day) can lead to episodes of light and mild diarrhea, nausea, hypersensitivity, and anal pruritus [175]. To the average healthy individual, these side effects may not be important to mention, but they may be a major disadvantage in individuals suffering from certain pathologic conditions.

### 6.2. Resveratrol Can Increase DNA Damage and Proteolysis

There is an abundance of published research that suggests vast health benefits of RE. Yet, adverse effects of RE in humans were also reported. These effects could be due to RE-increased ROS levels which may evoke proteolysis and DNA damage [176,177]. Although the actual biologically effective dose range of RE in vivo remains to be determined, it is important to differentiate the in vitro toxic concentrations (e.g. above 25 µM) from what may be the RE toxic dose in vivo. For instance, administration of an RE dosage of 20 mg/kg/day for 28 days to rats showed no adverse effects in the animals [178]. It is worth mentioning that this dose is 1000 times the amount consumed by a 70-kg human taking 1.4 g of *trans*-resveratrol/day. Using the same dosage, another study reported that RE enhanced sperm production in rats without any adverse effects [179]. However, when RE was administered, also to rats, for the same time, at a dosage of (0.3, 1.0, or 3.0 g/kg/day), aberrant expression of hepatic genes was noted, likely indicative of liver damage [180]. Four-week administration of RE at 0, 300, 1000, and 3000 mg/kg/day failed to induce adverse effects up to 300 mg/kg/day, while dosage of 1000 and 3000 mg/kg/day induced renal toxicity [35]. A significant increase in bilirubin levels was observed in rats administered with the 1000 (mg/kg)/day dose of RE, while 200 (mg/kg)/day in rats and 600 mg/kg/day in dogs did not cause adverse effects [168]. As such, it appears that RE needs to be administered in extremely high doses for it to elicit a significant toxic effect in vivo.

### 6.3. Human Trials with Resveratrol

RE intake has pleiotropic effects in humans [181]. Although it is generally well tolerated, some adverse effects including nephrotoxicity and gastrointestinal problems were reported in human subjects [182,183]. A 450 mg/day dose of RE was reported to be a safe dose for a 60-kg person [184]. However, RE at a dosage of 1000 mg/day or above was reported to inhibit cytochrome P450 isoenzymes such as CYP3A4, CYP2C9, and CYP2D6, while activating CYP1A2, thus leading to interactions with many other drugs [2]. Therefore, orally administered high doses (more than 1000 mg/day) of RE indicate differences in pharmacokinetics of concomitantly administered drugs. 

Despite the fact that RE seems to have beneficial antioxidant activity in human patients, there seems to be negative effects associated with RE intake on the metabolic status, endothelial health, inflammation, and cardiovascular markers in human patients [181]. In this context, a higher dose of RE (1000mg/day) was recently shown to elevate biomarkers of CVD risk (oxidized low-density lipoprotein (ox-LDL), soluble E-selectin 1 (sE-selectin 1), soluble intercellular adhesion molecule-1 (sICAM-1), soluble vascular cell adhesion molecule-1 (sVCAM-1), and total plasminogen activator inhibitor (tPAI-1) in overweight older adults, while lower doses did not have any effect on the same biomarkers [33]. These results are conformant with the hormetic effect of RE. Similarly, administration of 300 mg/day of RE failed to induce changes in the cognitive function, while the dosage of 1000/mg/day was able to selectively improve only the psychomotor speed (Trail Making Test) without affecting the other battery test [185]. The contradiction of the results is proposed to be due to the dose, varying gut microbiota, health status, and the bioavailability and pharmacokinetics of RE. Other causes for such differing results could be due to age, gender, lifestyle, the administration of RE with or without food, and the form of administration (caplet, tablet, powder, gel caps). As such, future research should conduct more uniform studies with similar study designs in order to eliminate the high level of unrelated variability [181].

Overall, RE is well tolerated in healthy individuals; however, not much research was conducted on patients with certain health conditions prior to administering RE. This could be one reason for the loss of a multiple myeloma patient in one clinical trial of RE intake [186]. In a phase II clinical trial on patients with refractory multiple myeloma, a daily dose of 5.0 g of RE was administered. Side effects of RE including nausea, diarrhea, fatigue, and renal toxicity might have caused the loss of this patient [186]. Hence, more in vivo research involving animal models of varying health status must be conducted prior to performing human studies to prevent the likelihood of the loss of patients during human trials. More clinical trials on RE are needed in humans before it can be considered for human therapeutic or preventative use. According to clinicaltrails.gov, several clinical trials were completed, but are yet to be published. When published, the results of such trials are expected to unravel more data into the use of RE in humans [187,188,189,190].

### 6.4. Resveratrol Impacts Cancer Onset: Clinical Studies

A role for RE in cancer was proposed, stemming mainly from in vitro studies looking at RE actions on cancer cells and their signaling pathways. There are, however, comparatively fewer studies that investigated the effect of RE treatment and its consequent outcomes on cancer patients in vivo. The main limitation in such studies is poor bioavailability of RE when orally administered [191]. Indeed, despite the fact that in vitro studies showed promising results, a number of in vivo studies failed to attribute beneficial effects to RE [58,192]. Poor RE pharmacokinetics could be a reasonable explanation for this phenomenon. For instance, it was found that 70% of a 25-mg oral dose of RE was absorbed in the intestines, but only trace amounts of RE were found in blood plasma [66]. Another reason could be the metabolism of RE into RE sulfate and glucuronide conjugates [66,193,194]. Gut microbiota is another variation that can lead to different in vivo effects of RE, whereby the gut bacteria can metabolize dietary RE into active and bioavailable metabolites [195,196]. To add, genetic background is another variation that can lead to variability of the enzymes involved in the sulfation and subsequent activation of xenobiotics, such as RE, in human subjects [197,198].

Administration of RE in animal cancer models showed variable effects: positive, negative, or completely neutral. This depended on the dosage, the tumor model itself, and the species of animals, amongst other variables like sex and strain of animals, method or timing of RE administration [199]. Thus, it is of utmost importance to homogenize the results of animal testing by conducting experiments with similar study designs prior to even attempting to study the effects of RE in humans. As a result, clinical evidence for the use of RE as an effective supplement in cancer prevention or treatment in humans is scarce. The first phase I clinical trial looking at RE as a therapy in human cancer patients was published in 2009 [200]. Patients with colorectal cancer had normal and cancerous intestinal mucosal samples biopsied at the time of diagnosis and 14 days following daily oral administration of RE (20 and 80 mg/day; *n* = 2 and 1, respectively) or grape powder (80 and 120 g/day; *n* = 3 and 2, respectively). Neither RE nor grape powder administration had an effect on Wnt signaling in the cancerous mucosa, but their supplementation resulted in decreased Wnt target gene expression in adjacent normal mucosa. Interestingly, the most significant effects were observed using low doses of grape powder. This led the authors to conclude that RE in combination with other compounds present in grapes could possibly be used to decrease the risk of colon cancer development by reducing Wnt pathway signaling. However, this treatment may not be as effective against an already established colon cancer [200].

In a similar context, it is known that the increase in insulin-like growth factor 1 (IGF1) and the decrease in IGF-binding protein 3 (IGFBP3) are correlated with tumor formation and metastasis. Supplementation of 2.5 g/day RE for 29 days significantly reduced IGF1 and IGFBP3 levels in plasma. This advances the notion that RE has chemopreventive activities [172]. Moreover, when healthy subjects were given 1.0 g of RE for four -weeks, lymphocyte counts and levels of enzymes involved in carcinogenesis and drug metabolism were favorably modulated with no significant adverse effects [201]. Hence, we believe that the safety of RE needs to be further investigated especially when considering the co-administration of RE with other medications.

**Figure 4:**
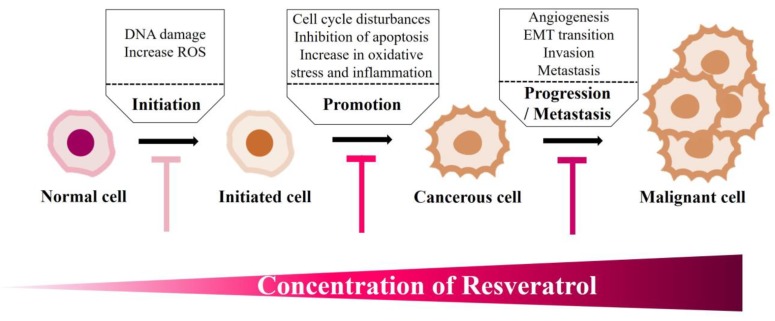
RE can affect all stages of carcinogenesis. RE can attenuate the various stages of cancer development, depending on its concentration. At low to moderate concentrations, RE, by acting as a chemopreventive agent, can block cancer initiation. This is achieved by suppression of spontaneous mutations and a reduction of cancer promotion that can lead to decreased tumor growth rate. At higher concentrations, RE can alter the late stages of carcinogenesis. By acting as a cytotoxic agent, RE can halt the progression and metastasis of cancer cells through the inhibition of angiogenesis and invasion of primary tumor cells. Hence, RE can be used to prevent cancer formation at its early stages or halt the progression and subsequent metastasis by acting as a cytotoxic agent [202,203,204,205,206].

Until now, the most promising use of RE, in cancer therapy, seems to be in cancer prevention rather than cancer treatment (Figure 3 and Figure 4). 

## 7. Enhancement of Pharmacokinetics Using Bio-Enhancers and Nano-Formulations May Overcome RE Adverse Effects

To enhance poor bioavailability and stability of RE, which consequently means lower need for intake of high RE doses and lower adverse effects, different kinds of drug carriers were tested and are being employed. These include nanoparticles, liposomes, and emulsions [207,208]. Solid lipid nanoparticles are novel drug carriers that can incorporate lipophilic drugs and improve their stability and bioavailability, water solubility, safety, bio-distribution, and biocompatibility [209]. Loading RE into poly-lactic-*co*-glycolic acid (PLGA) nanoparticles increased RE oral bioavailability up to 335.7%, in comparison to RE, alone following administration in rats [210]. Nanoparticle formulations even enhanced the therapeutic potential and efficacy of RE, especially its in vivo anti-cancer activities in several cancer types. RE was able to increase RE bio-distribution and decrease the tumor size of gliomas, as well as ovarian and colorectal cancers [211,212]. Further advancements in RE carrier delivery should help alleviate the harmful effects of high doses of RE, not only in cancer treatment but also in other diseases where RE showed therapeutic effects.

## 8. Resveratrol as a Complementary Therapy 

Evidence obtained from literature analysis points to the fact that the contradictory results from RE in vivo studies may be due to its poor bioavailability. However, RE poor bioavailability can be addressed by the employment of complementary therapy. Combining polyphenols with other bioactive components and micronutrients was reported to produce synergic therapeutic effects probably by enhancing bioavailability of polyphenols and expanding the metabolic effects of the combined agents [188,189,190]. Using their hydroxyl groups, polyphenols (Figure 2) can interact and associate with other compounds such as proteins and other nutrients, and this ultimately modulates their efficacy [187]. Polyphenol complexes may have a better stabilized chemical structure, enhanced solubility, and absorption into the small intestine in contrast to free polyphenols [213]. Polyphenol complexes have the ability to target multiple metabolic pathways, which could be another reason for employment of RE with other therapeutic combinations. Indeed, RE with different therapeutic combinations was reported to exert beneficial effects in different disorders and diseases, especially cancer [188,189,190]. This is despite the fact that RE can decrease the efficacy of certain drugs as mentioned above.

A tri-combination (TriCurin) of three polyphenols (curcumin obtained from spice turmeric, RE, and epicatechin gallate from green tea) was tested for its anti-cancer properties against human papillomavirus (HPV)-positive head and neck squamous cell carcinoma. When injected intratumorally in vivo, TriCurin was able to inhibit tumor growth by 85% as compared to the control, while, in vitro, it decreased cell viability, clonogenic survival, and tumor sphere formation, as well as significantly increased apoptosis [214]. Furthermore, TriCurin was able to decrease HPV16 E6 and HPV16 E7 and increase p53 protein levels [214,215]. In another study, relatively low doses of RE and epicatechin gallate were reported to inhibit casein kinase 2, which in turn can induce apoptosis in prostate cancer cells [216]. A combination of several polyphenols including RE, formulated as Cruciferex™, a compound obtained from cruciferous vegetables, was tested on human Fanconi anemia head and neck squamous carcinoma. The polyphenol mixture was able to significantly inhibit cell proliferation cell migration and matrix metalloproteinases (MMP) secretion [217].

Aside from achieving synergistic biological effects, combing vitamins with polyphenols was reported to stabilize, maintain, and support the activity of polyphenols, which could be an essential parameter for achieving the sought-after cooperative effects. A combination of RE and vitamin D3 was reported to enhance the estrogenic action of RE and its ability to modulate estrogen receptor (ER)-mediated transcription [190]. Such cooperative effects of RE and vitamin D3 were even reported in diabetic nephropathy. A combination of RE and vitamin D3 was shown to effectively reduce TNF-α and IL-6 expression when compared to individual drug treatments [218]. A combination of glucan, vitamin C, and RE demonstrated a strong anti-tumor potential by suppressing the growth of breast and lung tumors in in vivo models, which was superior to that of the individual agents [188]. These findings provide evidence that the combination of polyphenols, nutrients, and other agents with additive and/or complementary effects may be the way to achieve synergic actions in the face of cancer and other diseases that need to be targeted at different molecular pathways.

## 9. Current Concerns and Recommendations 

The biological effects of RE, as well as its in vitro and in vivo outcomes, appear to be strongly associated with a hormetic effect where RE low doses usually are associated with beneficial effects while high doses usually have a toxic effect [44]. In this regard, evidence suggests that RE’s hormetic property may be due to its dose-associated biphasic effect on the cellular redox state, which was reported to be antioxidant at low doses and a pro-oxidant at high doses [38,43,44,63]. As such, there are concerns that studies on the compound mostly focused on the short-term outcomes of RE intake. Given that we suggest that many of the controversial results present in the literature may be due to this hermetic aspect, it is suggested that RE dosage and RE interaction with the redox state of the environment appear to be of primary importance; especially when precise redox modulation is needed to allow a physiological function or to promote a deleterious effect. Other aspects related to RE controversial data appear to be differences in the characteristics of the enrolled patients, RE doses used, and the duration of RE supplementation; therefore, more extensive studies in more complex models are warranted in order to validate the current findings. 

Notwithstanding the substantial number of human and animal studies that support the beneficial and protective properties of RE [60,181,219,220,221,222], there are not enough clinical studies that report on RE’s harmful effects, which are indeed full of controversy. Moreover, the molecular mechanism of RE action needs to be better identified. All of these contradictions call for an urgent need to appraise and investigate the adverse outcomes of this compound despite its documented benefits. Above all, the high level of variability among all the different studies calls for a more uniform design of clinical trials to properly investigate the effects of RE and define its mechanisms of disease therapy and prevention.
